# Novel System for Bite-Force Sensing and Monitoring Based on Magnetic Near Field Communication

**DOI:** 10.3390/s120911544

**Published:** 2012-08-24

**Authors:** Andres Diaz Lantada, Carlos González Bris, Pilar Lafont Morgado, Jesús Sanz Maudes

**Affiliations:** 1 Mechanical Engineering Department at Universidad Politécnica de Madrid, c/José Gutiérrez Abascal 2, 28006 Madrid, Spain; E-Mail: plafont@etsii.upm.es; 2 Conectivity Group, Higher Technical School of Telecommunication Engineering, Universidad Politécnica de Madrid, Av. Complutense 30, 28040 Madrid, Spain; E-Mails: cgonzale@etsit.upm.es (C.G.B.), jsanzmau@etsit.upm.es (J.S.M.)

**Keywords:** wireless communication, magnetic field communication, biomedical telemetry, biomedical monitoring

## Abstract

Intraoral devices for bite-force sensing have several applications in odontology and maxillofacial surgery, as bite-force measurements provide additional information to help understand the characteristics of bruxism disorders and can also be of help for the evaluation of post-surgical evolution and for comparison of alternative treatments. A new system for measuring human bite forces is proposed in this work. This system has future applications for the monitoring of bruxism events and as a complement for its conventional diagnosis. Bruxism is a pathology consisting of grinding or tight clenching of the upper and lower teeth, which leads to several problems such as lesions to the teeth, headaches, orofacial pain and important disorders of the temporomandibular joint. The prototype uses a magnetic field communication scheme similar to low-frequency radio frequency identification (RFID) technology (NFC). The reader generates a low-frequency magnetic field that is used as the information carrier and powers the sensor. The system is notable because it uses an intra-mouth passive sensor and an external interrogator, which remotely records and processes information regarding a patient's dental activity. This permits a quantitative assessment of bite-force, without requiring intra-mouth batteries, and can provide supplementary information to polysomnographic recordings, current most adequate early diagnostic method, so as to initiate corrective actions before irreversible dental wear appears. In addition to describing the system's operational principles and the manufacture of personalized prototypes, this report will also demonstrate the feasibility of the system and results from the first *in vitro* and *in vivo* trials.

## 1. Introduction

Intraoral devices for bite-force sensing and measurement have several applications for odontology and maxillofacial surgery, as an additional method for assessing the degree and for monitoring of dental and occlusal pathologies and for assessing the functional state of the masticatory system [1]. It is also used for the evaluation of post-surgical evolution and for comparing alternative treatments and their influence on temporomandibular disorders [2]. Because the information derived from such measurements can be used efficiently in clinical tasks, the development of precise and repetitive sensing systems is especially valuable [3]. Several devices (and related patents) have been developed in the past two decades for the aforementioned tasks, allowing intraoral (almost always between occlusal surface of teeth and between upper and lower teeth) bite-force sensing using different mechanical and electronic sensing principles, the most relevant of which are mentioned below.

The use of extensiometric gauges has been reported and validated *in vivo* using animal models, such as primates [4], pigs [5] and dogs [6]. In other cases, several devices have been used to assess the degree of patient satisfaction with dental prostheses, both fixed and removable, in order to validate therapeutic solutions and to propose additional improvements [7–9]. Among the most important studies linked to intraoral bite-force sensing in humans are those related to the study of bruxism activity and temporomandibular joint pathologies [10–12].

Bruxism is a health problem that involves grinding or tightly clenching the upper and lower teeth. Both the grinding and the sliding lead to dental wear and produce a noise during the night that is sufficiently loud to disturb the sleep of anyone nearby, among other symptoms described further on. The phenomenon was introduced in the dental literature as “bruxomania” in 1907, by Pietkiewicz, who described the habit of teeth grinding. The term “bruxism” was introduced by Frohman in 1931 and, in 1936, Miller proposed the use of the term “bruxomania” for daytime grinding and “bruxism” for nighttime grinding. The terms “traumatic neuralgia”, “Karolyi effect” and “occlusive habit neurosis” have all been used to refer to some form of teeth grinding or clenching.

The term bruxism is mainly used when the duration and intensity of clenching or grinding activities have a bearing on dental wear and lead to the development of temporomandibular joint (TMJ) problems. However it is important to point out that everyone subconsciously clenches his or her teeth at some time, even healthy population, due to different tooth damages, corporal pains or social conflicts, and this may also be considered bruxism activity [13].

According to studies by the Canadian Sleep Society [14–16], nocturnal bruxism affects 8% of adults and 14% of children. A decrease in the population affected is apparent with age, with around 3% of individuals over 60 being affected. However, for some researchers [17,18] prevalence is around 25%. In terms of gender differences in the affected population, there is no general agreement data.

Main symptoms include lesions to the teeth; problems in the muscles, tissues and other structures surrounding the jaw; headaches; ear pain; reduced neck motility; and, sometimes, irreversible disorders of the jaw joints [13]. All of these symptoms are also collectively described as temporomandibular joint disorders or craniomandibular dysfunction pain syndrome.

Measurements of intraoral bite-force become especially relevant when related to the study of bruxism, especially as a complement for polysomnographic diagnosis, which still is the most adequate procedure [15] for monitoring the evolution of patients and comparing different possible treatments. Special attention should be paid to results from some studies [10–12] that have tried to offer a quantitative definition of bruxism. Results from Nishigawa's study on the bite force produced during bruxism events suggested values as high as 1,100 N, exceeding the maximum voluntary bite force. Pressures on teeth surfaces can reach 40 MPa, sufficient to cause alarming levels of wear and even tooth breakage. In terms of the duration of bruxism events, an average of around 7 seconds has been reported [10,11]. When developing sensors, it is important to distinguish bruxism events from mioclonus or rapid contractions (<0.5 s) of the jaw muscles [11].

One of the main problems associated with the traditional diagnosis of bruxism is that it is frequently made when the teeth are already highly worn and the prognosis of the illness is more severe [19]. Bruxism activity can also be recorded by an electroencephalogram (EEG), as well as by means of electromyography (EMG) and surface electromyography (S-EMG). Video cameras are also used to distinguish between bruxism events of the mioclonus and rapid contractions (<0.5 s) of the jaw muscles [20]. In order to provide additional information for understanding the characteristics of bruxism disorder, as additional information for conventional diagnosis, and to compare different treatment alternatives of bruxism, our research team proposes the use of novel instrumented splints for detecting and recording the intensity and duration of bruxism events.

Explained below are the design, manufacture and trials of a constructed splint, for the monitoring of bruxism activity, which also includes additional telemedicine possibilities, such as patient remote monitoring and control. The reduced-size sensing device allows obtaining an intra-mouth device, which does not cause important alterations of the patient's bite and promotes the quality of final measurements. The use of a wireless external interrogator/reader also allows the collection of the desired information in a more convenient way, when compared to previous proposals from our team [21,22] and to the devices used in other studies [23,24].

## 2. Design of Bite-Force Sensing and Monitoring System

### 2.1. Principle of Operation

In this work, we propose a system and explain the development of a prototype that uses a wireless connection between a passive force sensor, located within a splint in the mouth cavity, and an active external unit that energizes the sensor and permanently records all force measurements. This system allows for permanent occlusal force measurements. The prototype uses a magnetic field communication scheme similar to low-frequency radio frequency identification (RFID) technology (NFC). The reader generates a low-frequency magnetic field that is used as the information carrier and powers the sensor.

Unlike other RF signals, the low-frequency magnetic field is not absorbed by biological tissues. This may be an advantage over Bluetooth-based systems [21,22]. However, the communication range is limited to a few centimeters in order to achieve reasonable amplitude values for the excitation signals.

A low-frequency (125 kHz) magnetic carrier field was chosen because it offers good penetration and is not absorbed by biological tissue. Additionally, the inductive coupling of the carrier field allows us to energize passive components and ensures that the transmitting coils and other components will be sufficiently small for intraoral measurement.

Our team is not aware of any rigorous studies of the effects of the low frequency magnetic field in human tissue. Nevertheless, a high intensity low frequency magnetic field is commonly used in passive RFID scanners in consumer retail contactless payment systems, so we initially consider it biologically safe, although losses in metallic fillings need to be considered. However additional long-term testing in animal models should be accomplished before obtaining a product that could be produced on an industrial scale. RF communication suffers from higher absorption in biological tissue, and it does not allow the sensor to be externally powered, thereby requiring the use of a battery. The size of the components is proportional to the operation frequency.

Like RFID, the basic system is composed of an interrogator and a passive sensor. The term passive indicates that the sensor does not use a battery. The interrogator (reader) is usually composed of a microcontroller-based unit and an analogue front-end. The analogue front-end generates a low-frequency (125 kHz) magnetic field that induces a voltage within a tuned LC circuit in the power subsystem of the sensor. This voltage is rectified and used to power the rest of the sensor. This tuned LC circuit is not the same as the LC circuit that is used to communicate with the reader because energy must be permanently supplied to the sensor for it to operate, while the communication module can be independently tuned and detuned.

The small variation produced in the field by the modulation does not affect the energizing module of the sensor. The sensor is only powered when there is sufficient induced voltage in the coil, which implies a maximum distance requirement for the reader. When the sensor is energized, it activates a low power oscillator with an oscillation frequency that is directly related to the resistance value of a force-resistance transducer. A force-dependent oscillator was chosen because it permits continuous monitoring if the interrogator is active and it draws less power in continuous operation than a micro-controlled-based unit that samples, converts and digitizes the readings. In order to transfer the data to the reader, the output of the oscillator is used to bypass a capacitor within an LC tank that is tuned at the frequency of the interrogating field, so that the oscillator tunes and de-tunes the LC circuit at the force dependent oscillating frequency, producing what is commonly termed backscattering or load modulation.

This modulation is sensed at the interrogator as a tiny amplitude modulation (typically around 60 dB), which is recorded using an envelope detector and a locking amplifier. In this manner, the frequency of the detected signal equals the frequency of the oscillating sensor, which depends on the applied force. Frequency modulation was chosen instead of amplitude modulation to make the system less sensitive to relative head movements, which imply distance variations and, correspondingly, undesired amplitude modulation. By contrast, frequency is not affected if the sensor is within communication range.

The oscillator frequency range for the expected transducer values must be selected to match the frequency response of the reader. The demodulated frequency signal is used to evaluate the sensed bite force. The sensor is designed to be placed inside a splint located in the mouth, without remarkably affecting the size of conventional Michigan-type occlusal splints and main types of mouthguards.

**Figure 1.**
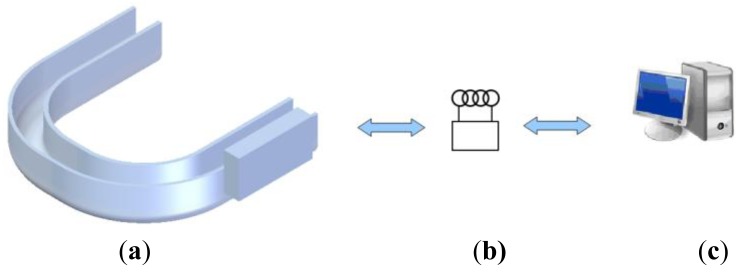
(**a**) Intraoral splint with passive force sensor. (**b**) External wireless interrogator. (**c**) Subsequent data delivery to PC for additional studies.

Although a patent for the described system [25] with reference number OEPM P200900875 [26] has been granted by the Spanish Patent and Trademark Office (the University holds the rights), the authors do not intend to develop a commercial product at this time; moreover, significant more funding and extensive *in-vivo* testing using animal models would be mandatory in such a case. A diagram of the components and operating scheme is shown in Figure 1.

### 2.2. Intraoral Passive Bite-Force Sensor

**Figure 2.**
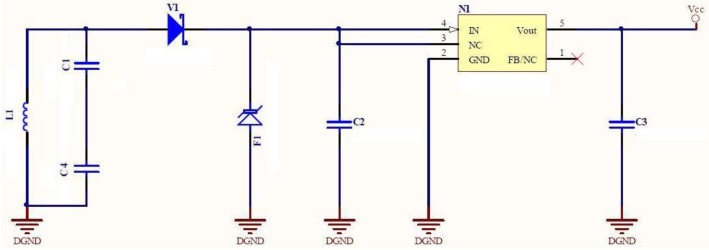
Energizing subsystem of the sensing unit.

The passive sensor, which is the component that must eventually be integrated into the splint, is composed of two different subsystems:

- The first subsystem is a power subsystem composed of a tuned LC circuit, a Schottky diode rectifier and a low power, low dropout regulator (f.i., MC78LC30) that provides a constant DC voltage with two capacitors at its input and output. This approach avoids using a battery within the mouth. The values of the LC circuit are chosen so that it resonates near 125 kHz.
  Ideally:
  $$\sqrt{LC}=2\pi \;{\text{f}}_{0}=2\pi \;125\;\text{kHz}$$
  Figure 2 depicts the schematic of the energizing subsystem. Subsystem (I) energizes subsystem (II) given sufficient interrogation field intensity.
- The second subsystem is a force-sensitive oscillator, which is composed of a low power relaxation oscillator (LMC555 from National Semiconductor) and a few passive components, including a customized for short length (25.4 mm long) commercial force to resistance transducer (ZFLEXA201-100 from Tekscan), which is 0.2 mm thick and features a sensing area of about 0.78 cm^2^. Its low thickness is especially appropriate for being integrated in a intraoral device and for not affecting patients' bite performance dramatically. Such thickness is similar to that from other film sensors (normally piezoelectrics) previously used in related research [27].

According to manufacturer (Tekscan) this resistance transducer is constructed using two layers of substrate. This substrate is composed of polyester film (or polyimide in the case of high-temperature applications). On each layer, a conductive material (silver) is applied, followed by a layer of pressure-sensitive ink. Adhesive is then used to laminate the two layers of substrate together to conform the sensor. The silver circle on top of the pressure-sensitive ink defines the “active sensing area”. Silver extends from the sensing area to the connectors at the other end of the sensor, forming the conductive leads. The sensor acts as a variable resistor in an electrical circuit. When the sensor is unloaded, its resistance is very high (greater than 5 MΩ). When a force is applied to the sensor, the contact between conductive particles of the pressure-sensitive ink is promoted and the resistance decreases. Additional information on the working principle and conditioning proposals can be obtained from specification sheets available at Tekscan's website (www.tekscan.com, last access 1 August 2012).

**Figure 3.**
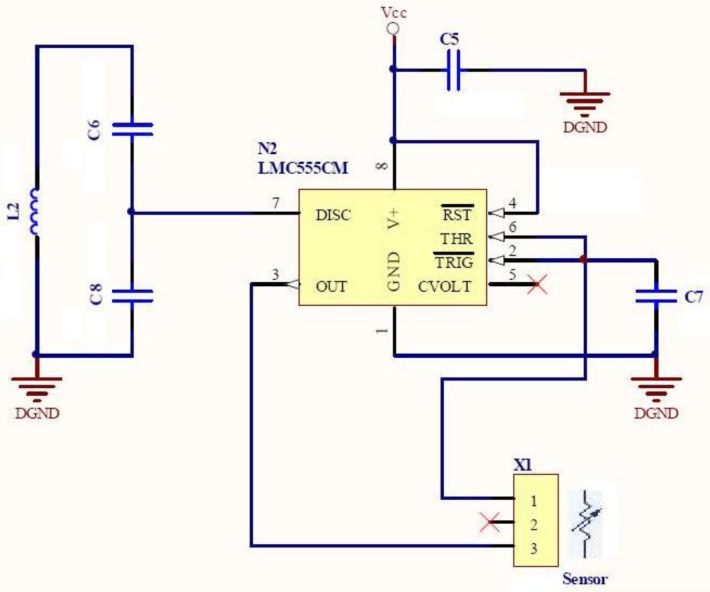
Force-sensitive oscillator subsystem of the sensing unit.

Such a transducer must be placed in the splint, where it converts the occlusal force into a resistance, which determines the oscillation frequency. When force is applied to the transducer, its resistance decreases and such decrease produces a frequency shift in the oscillator. The output of the relaxation oscillator drives a tuned LC circuit, which is tuned and de-tuned at the oscillation frequency. The sensor oscillation frequency range of this component is chosen to match the response of the filter located at the reader element, and the maximum oscillation frequency is selected to match the minimum resistance of the force transducer, which presumably happens when a bruxism event is occurring. When operating, this component consumes about 250 μW. Figure 3 shows the schematic of the force-sensitive oscillator.

**Figure 4.**
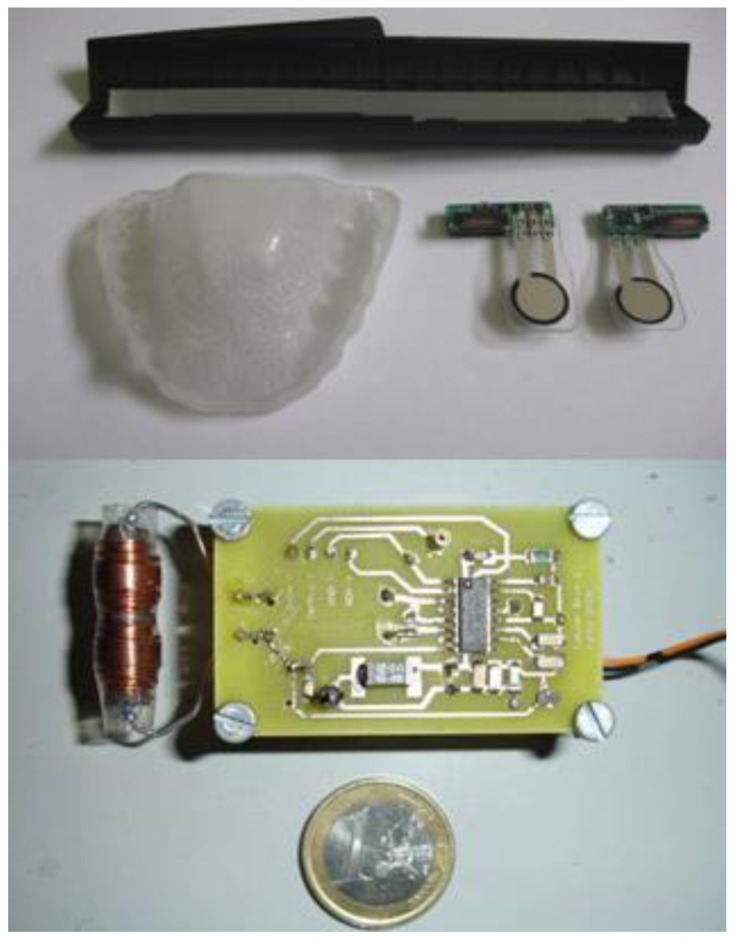
Splint, passive sensors with transducers and photopolymerizable resin for encapsulation (**upper image**). View of the external interrogator prototype (**lower image**).

Figure 4 (upper panel) shows two prototypes of the passive force sensor. The implemented sensing unit made up of a printed circuit that is 2 cm long, 5 mm wide and 5 mm thick and a force to resistance transducer, which is trimmed to a length of about 25.4 mm, which is soldered to the printed circuit so that it appears as a protruding tongue. This unit is integrated in the splint.

### 2.3. External Interrogator

A reader like the one used in this research is typically composed of a microcontroller and an analogue front-end unit. This unit generates the field that energizes the passive sensor and detects the modulated signal that is produced at the excitation coil by the tuning and de-tuning of the sensor tank circuit. In our first implementation, we have only used a front-end unit that, once powered, permanently generates the magnetic field using a series LC circuit. The microcontroller permits control of the excitation activity duty cycle of the front-end unit and readout of the demodulated signal. The demodulated output provided by the front-end unit was monitored using an oscilloscope in our case. In the prototype, we used an (EM4095) analogue front-end with external passive components.

The components were selected to generate a 60 Vpp at the excitation coil. An antenna current of 230 mArms corresponds to this value, which is below the 300 mArms limit listed in the specifications. A supply current of 175 mA was measured under the operating conditions with a voltage of 5 V. This value results in a power dissipation of 875 mW, which corresponds to about 200 mW in the chip and 700 mW in the external current limiting resistor located in the excitation tank. The chip temperature increase is about 15 °C, which is less than the specified maximum temperature value (100 °C) at normal room temperature. A set of four standard rechargeable AA batteries lasts about 12 hours if the unit is permanently in the ‘on’ state. The values of certain components within the circuit determine the filtering behavior of the demodulator. All components were selected to maximize sensitivity at a frequency of nearly 2 kHz, which corresponds to the force threshold applied to the transducer during bruxism events. Figure 4 (lower panel) shows the interrogator/reader prototype used to evaluate the system.

### 2.4. Prelimminary *In Vitro* Calibration

Prototype passive sensor behavior was characterized using a workbench based on pneumatic muscles (Festo MAS-20-160N-AA-MC-K) capable of applying bite forces in the range of 0–1,400 N. The measurement range was selected because the occlusal force during a bruxism event is expected to reach around 1,000–1,200 N, in accordance with the values that have been measured during events in controlled environments [22,27]. The final value will depend on each specific patient.

To obtain the desired calibration curve, the transducer of the sensor was placed on the workbench between two metallic pieces. Force was applied using the pneumatic muscle, while the sensor was activated by the interrogator and each force level was repeated three times. The output demodulated signal provided by the reader was measured using the screen trace of an Agilent Infiniium 54832D oscilloscope, together with a USB data acquisition board, Measurement Computing model LS 1208.

**Figure 5.**
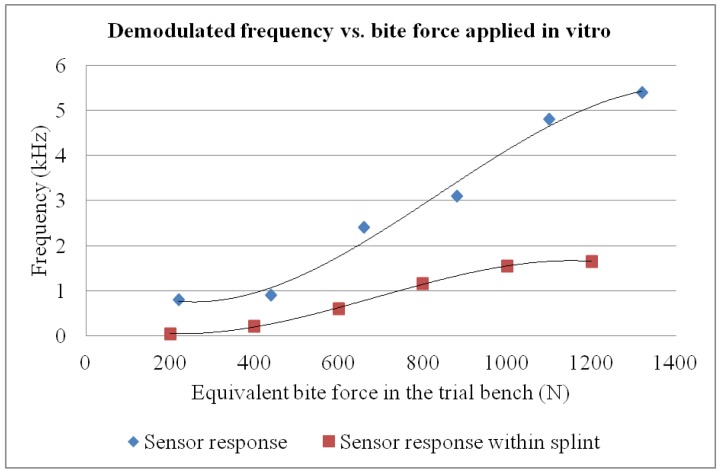
Sensor calibration curve before encapsulation and once encapsulated within the splint.

Applied force can be calculated as a function of the supply pressure applied by taking into account the geometry of the workbench and the response characteristics of the muscle. Figure 5 illustrates the results and for each force level studied a standard deviation below 5% was measured among repetitions.

The calibration curve of the sensor, before encapsulation within the intraoral protection splint, proves to be adequately linear in the measurement range of typical bruxism events (350–1,000 N). The observed communication range is about 5 cm if the sensor is energized using a constant power supply (*i.e.*, using an external supply or battery). Nevertheless, the limited communication range can be considered an advantage because it avoids interference with other devices.

It is important to note that final sensor encapsulation within the splint changes measurement, because the photopolimerizable resin used (see Section 3) absobs the applied force, so a final calibration step, once the sensor is included within the splint, is needed for finally relating measured frequency shift and actual bite force. For such final calibration an *ad hoc* workbench can be employed, with pneumatic muscles acting on personalized plaster models reproducing the teeth of the simulated patient, which finally act on the splint, as has already used for our previous developments [22,27]. The effect of sensor encapsulation within the splint can hence be assessed and is represented below in Figure 5 as final device characterization step.

## 3. Prototype Manufacture

The idea of including the passive force sensors within an intraoral splint responds to a need to locate the transducer in a fixed location between the teeth of the patient, so as to obtain repeatable measurements. At the same time, such polymeric splints help to protect and isolate the system. The manufacturing process is explained below.

First, two silicone moulds of the upper and lower teeth of the patient are obtained by rapid form copying. Plaster models of a patient's teeth are subsequently obtained by casting with the help of the aforementioned silicone moulds. The base layer of the splint is obtained by vacuum/pressure thermoforming against the plaster models using (Erkodur) thermoplastic, 1.5 mm-wide polymeric discs. This base layer gives structural support to the whole system and after careful polishing, can be secured to the upper teeth of the patient. The personalized design provides additional comfort. The electronic components are encapsulated and adhered to the base layer of the splint with the help of a Kuss Dental Kit.

**Figure 6.**
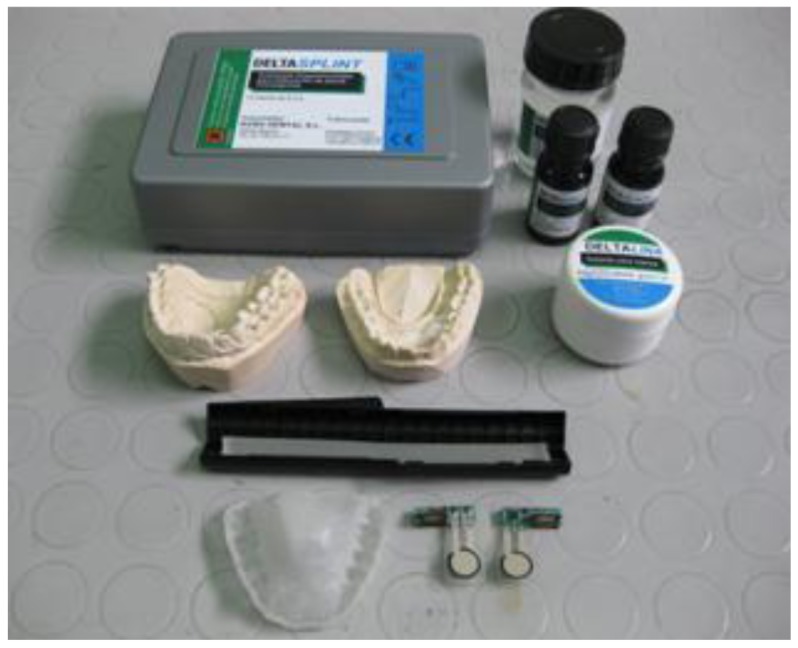
Components for manufacturing the intraoral splint.

**Figure 7.**
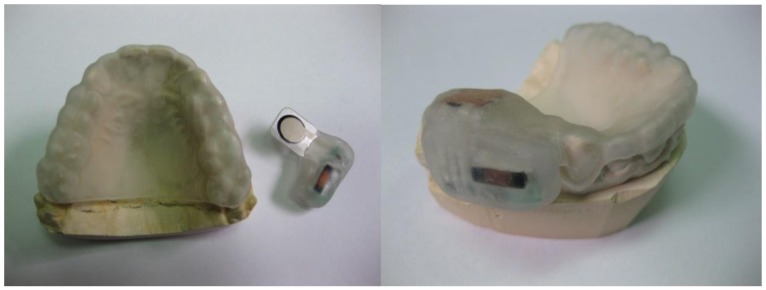
Splint prototype manufacturing process: Instrumentation of the intra-oral splint.

Such splint manufacturing kits include the photopolymerizable resin “Delta Splint”, which can be conformed manually to the desired forms, glued to the base layer with the help of “Delta Bond” and finally cured by UV exposure. Using the “Delta Splint” resin, the passive force sensor is first encapsulated. After curing it is bonded to the base layer so that it can be positioned in the vestibule of the mouth. Additional protective layers of photopolymerizable material can be included to enhance results. The step-by-step manufacturing process is shown in Figures 6 and 7.

It is important to note that the use of the mentioned photopolymerizable resin allow encapsulating at room temperature, what proves to be very positive for protecting the electronics from non-desired damages that usually appear when using high-temperature processing stages. We have not appreciated any effect due to the resin shrinkage during curing, as the layer of resin applied is very thin (around 1 mm), although further specific assessment of such influence may be of interest if the system is encapsulated in other biodevices requiring for instance spherical or cylindrical encapsulations.

The exposed process is also noteworthy due to its versatility, helping to find the most ergonomic location for the intra-oral components, and is especially well-suited for research studies related to pre-commercial devices, in which final distribution of components is not yet defined. Regarding industrialized manufacturing processes previous studies from our team have also analyzed the use of semi-automatic thermoforming between several layers of acrylic thermoplastic polymeric discs for encapsulation [22], valid for the production of short and medium series, although not so versatile for prototype manufacture and preliminary validation stages.

Final device size is similar to that of recent devices for intra oral monitoring [23,24] and the *in vivo* trials, explained further on, help to validate our approach in a preliminary way, before carrying out a more exhaustive clinical study for analyzing additional factors of influence such as, sex, age, professional activity, combined pathologies, among others, also with the patients during their sleep. To our knowledge it is the first intraoral wireless and battery-less intraoral devices with these diagnostic capabilities, what helps also to enhance usability and final device safety.

## 4. *In Vivo* Trials for Validation

**Figure 8.**
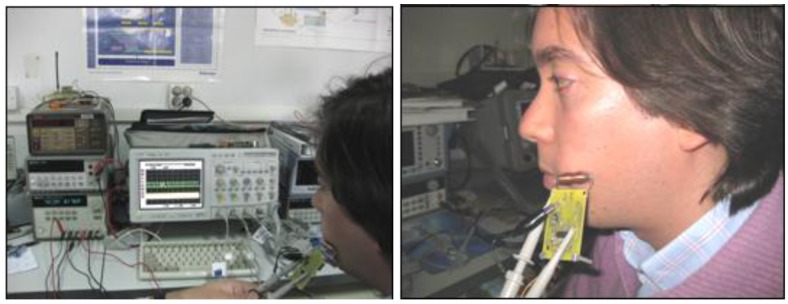
*In vivo* validation trials.

After the bite-force sensing system prototype was manufactured, *in vivo* trials were carried out to validate its capabilities and advantages compared with existing devices, as well as to evaluate principal required improvements. Figure 8 shows the *in vivo* validation, including the intraoral constructed splint from Figure 7 (located inside researcher's mouth, so it cannot be seen) and the external interrogator from Figure 4, place on the researcher's face and connected by cable to an Agilent Infiniium 54832D oscilloscope as the frequency signal detector, in which the frequency of the signal was read from the screen output.

The external interrogator was always placed against the face of the patient (30 year old researcher without occlusal problems) so that the relative position between the passive sensor and the interrogator would not change. It is important to note again that no wired connection was used between the intraoral instrumented splint and the external interrogator, as the system works by wireless magnetic field communication. The cables seen in Figure 8 just connect the external interrogator to the battery package and to the oscilloscope for measurement recording.

**Figure 9.**
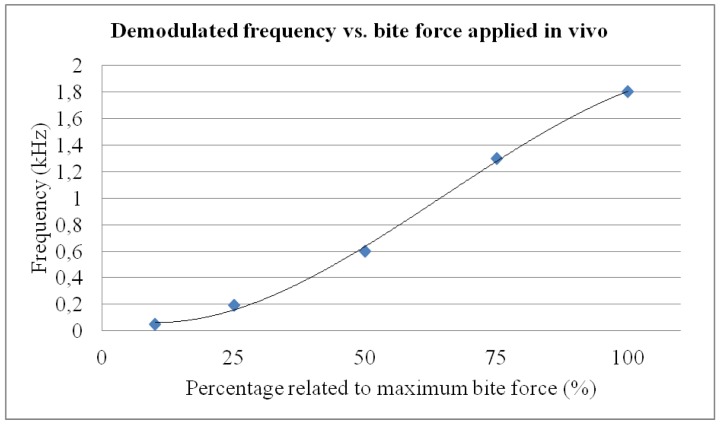
Results summary. Frequency *vs.* applied force (related to maximum bite force).

The system proved to be reliable and accurate with a communication range of around 3 cm, even though the sensor and reader operated through human flesh. A higher range can be achieved using a higher excitation. This range cannot be easily obtained using other wireless approaches, such as Bluetooth/ZigBee. The main results are summarized in Figure 9 and explained further on.

During the *in vivo* trials, bruxism events were deliberately simulated to verify the capability of the system to quantitatively assess events of different magnitudes. The researcher was first asked to consciously bite with maximum force. Afterwards, he was requested to apply bite forces of different magnitudes relative to the initial bite force applied (75%, 50%, 25% and 10%) to assess system behavior within the mouth of a person and its quantification ability, as shown in Figure 9. Such *in vivo* trial was intended to provide a conscious simulation of different bruxism events, as well as to assess the mechanical stability and performance of the prototype inside patient's mouth. Each bite force level was repeated five times and maintained for 5 seconds, with a rest interval of 10 seconds between each measurement, so as to verify repeatability of the measuring system. For each bite force level, final standard deviation obtained was below 15%, what we consider adequate for prototype quality validation and taking into account the limitations of *in vivo* probing, highly useful for assessing the device integrity and response in real working conditions, but somehow limited when trying to simulated similar events several times. With the calibration curve of the constructed splint (see Figure 5), such demodulated frequencies can be related to actual bite forces.

In addition the transversal component of force performed during bruxism events was also simulated, but the system did not response in a predictable way, as the transducer used here is designed for the measurement compressive stresses. A measurement of both vertical and horizontal forces can be obtained by using a different kind of transducer, such as piezoelectric polymers (*i.e.*, PVDF, due to its non-zero value of d_33_, d_31_ and d_32_ piezoelectric coefficients), as our previous studies have shown [27]. However the use of polymeric piezoelectric sensors requires more complex conditioning, including a charge amplifier, and signal is less robust to the influence of movements and external signals. In addition the piezoelectricity of these polymers is consequence of their being ferroelectric and, therefore also pyroelectric, so the final signal is also highly influenced by temperature changes [28].

Future trials should address a possible combination of different kinds of sensors for improving the diagnostic capabilities of the system, specially focusing on providing additional information about horizontal forces (interesting for transversal bruxism) and further device miniaturization should be pursued to allow more comfortable recording of bite force of bruxism patients during sleeping. Our advice would be to change the current external reader morphology, using vertical axis flat excitation coil, which could be located like a patch on patient's face. Integration of components would significantly reduce external reader's size and additionally improve the comfort of all-night monitoring.

We also noted that, due to the additional protection of the transducer provided by the splint, the values of the demodulated frequencies measured *in vivo* are smaller than those obtained with the initial *in vitro* calibration using the trial bench. As instrumented splints can be manufactured using different polymeric materials, with different elasticity, the behavior of the system after manufacture should always be calibrated *in vitro*, using for instance a pneumatic workbench [22,27], in accordance with the desired personalized approach.

In any case, linearity of the system is especially favorable in the conventional bruxism event range (from 30% to 90% of maximum bite force, *i.e.*, around 300 to 1,300 N). The results of our trials with a personalized splint show that it is possible to detect bruxism events of different intensities and durations, which, combined with the ability to record and store the data, converts the system into a “Holter” for bruxism diagnosis/monitoring and into an additional tool for evaluating clenching forces or bruxism activity.

## 5. Conclusions

This paper has focused on the complete development of a splint to assess intraoral clenching forces, as an aid or additional resource for diagnosing bruxism and other occlusal pathologies. The system includes an active interrogator/reader and a passive sensor that can be used to detect and record bruxism events. The system uses a low-frequency magnetic field to energise the passive sensor, located within the splint, and to detect information about the force magnitude before transmitting it via a communications protocol that is similar to RFID. The passive sensor extracts the energy that it needs to operate from the excitatory field and converts the sensed force into a frequency signal that is sent wirelessly to the reader using load modulation. Additional remote monitoring activities can be directly implemented *i.e.*, via mobile phone.

A prototype of the passive sensor/active reader has been built to evaluate and characterize the behaviour of the sensing system. Our results indicate that the sensor can be used to continuously monitor force as a function of time, although it can also be enabled/disabled periodically by a controller. The prototype is only intended to evaluate the viability of the approach; final integrated devices may be the size of a tooth. The system, to our knowledge, is unique in its use of magnetic fields to energise the passive force sensor, which allows for permanent function and eliminates the requirement for intraoral batteries. Hence, the proposed system is much safer than previous instrumented splints with communication capabilities that demand an intraoral energy supply.

Additionally, we highlight the possibility of obtaining a device, whose measurements will provide additional information for diagnosis of bruxism and conservative treatments can be taken before the appearance of irreversible dental wear.

The first *in vitro* and *in vivo* trials to date have served to demonstrate the feasibility of the system in accordance with our proposed objectives. Finally, it should be emphasized that a similar working principle can be applied to the development of several biomedical and biomechanical devices that can benefit from force measuring capabilities and wireless communication, even working through patient's flesh or tissues, such as instrumented intelligent prosthesis or health monitoring systems.

Future efforts will be directed to a more systematic *in vivo* study, in order to assess the actual clinical effectiveness of the proposed device, aiming at an earlier diagnosis of craniofacial and occlusal disorders, including bruxism.
